# In Depth Viral Diversity Analysis in Atypical Neurological and Neonatal Chikungunya Infections in Rio de Janeiro, Brazil

**DOI:** 10.3390/v14092006

**Published:** 2022-09-10

**Authors:** Maria Celeste Torres, Fatima Di Maio, David Brown, Moira Spyer, Eleni Nastouli, Patrícia Brasil, Ana Maria Bispo de Filippis

**Affiliations:** 1 Laboratório de Flavivírus, Instituto Oswaldo Cruz, Fiocruz, Rio de Janeiro 21040-900, Brazil; 2 Departament of Neonatology, Universidade Federal do Estado do Rio de Janeiro, Rio de Janeiro 20270-004, Brazil; 3 Advanced Pathogen Diagnostics Unit, University College London Hospitals NHS Trust, London NW1 2PG, UK; 4 Department of Infection, Immunity and Inflammation, UCL GOS Institute of Child Health, University College London, London WC1N 1EH, UK; 5 Laboratório de Doenças Febris Agudas, Instituto Nacional de Infectologia Evandro Chagas, Rio de Janeiro 21040-360, Brazil

**Keywords:** chikungunya virus, atypical manifestations, placenta, blood–brain barrier, intrahost diversity

## Abstract

Chikungunya virus (CHIKV) is an arthropod-borne virus (arbovirus) transmitted by Aedes mosquitoes. The human infection usually manifests as a febrile and incapacitating arthritogenic illness, self-limiting and non-lethal. However, since 2013, CHIKV spreading through the tropics and to the Americas was accompanied by an increasing number of cases of atypical disease presentation, namely severe neuropathies and neonatal infection due to intrapartum vertical transmission. The pathophysiological mechanisms underlying these conditions have not been fully elucidated. However, arbovirus intrahost genetic diversity is thought to be linked to viral pathogenesis. To determine whether particular viral variants could be somehow associated, we analyzed the intrahost genetic diversity of CHIKV in three infected patients with neurological manifestations and three mothers infected during the intrapartum period, as well as their babies following vertical transmission. No statistically supported differences were observed for the genetic variability (nucleotide substitutions/gene length) along the genome between the groups. However, the newborn and cerebrospinal fluid samples (corresponding to virus passed through the placenta and/or the blood–brain barrier (BBB)) presented a different composition of their intrahost mutant ensembles compared to maternal or patient serum samples, even when concurrent. This finding could be consistent with the unidirectional virus transmission through these barriers, and the effect of selective bottlenecks during the transmission event. In addition, a higher proportion of defective variants (insertions/deletions and stop codons) was detected in the CSF and maternal samples and those were mainly distributed within the viral non-structural genes. Since defective viral genomes in RNA viruses are known to contribute to the outcome of acute viral infections and influence disease severity, their role in these atypical cases should be further investigated. Finally, with the in silico approach adopted, we detected no relevant non-conservative mutational pattern that could provide any hint of the pathophysiological mechanisms underlying these atypical cases. The present analysis represents a unique contribution to our understanding of the transmission events in these cases and generates hypotheses regarding underlying mechanisms, that can be explored further.

## 1. Introduction

Chikungunya fever is a disease caused by the chikungunya virus (CHIKV), an arbovirus that belongs to the *Togaviridae* family, Alphavirus genus [1]. It is primarily transmitted to humans by Aedes mosquitoes and infection is usually characterized by fever, rash, and severe persistent polyarthralgia [1]. Since 2004, CHIKV has spread extensively through the tropics and to the Americas in 2013. It has caused explosive outbreaks with atypical clinical presentations increasingly recognized [2,3,4]. Complications of the nervous system are the most common severe complication of chikungunya infection (approximately 0.1% of symptomatic cases) [5,6,7]. Cardiovascular, renal, respiratory, hepatic, gastrointestinal, and adrenal are reported, and although infrequent, they can be severe, with high morbidity and mortality rates [5,8,9].

The most common neurological disorders associated with CHIKV infection are encephalopathy and other central nervous system (CNS) disorders, followed by pure peripheral nervous system disorders such as Guillain–Barré syndrome, and less commonly mixed pathogenesis such as encephalomyelopathies or myeloneuropathy, affecting multiple parts of the nervous system [5]. The pathophysiological mechanisms by which CHIKV affects the nervous system have not been fully elucidated, and whether this is due to direct damage following viral replication or indirectly by triggering an immune-mediated acute inflammatory damage. Indeed, both mechanisms are not mutually exclusive and could be both involved in the CNS pathogenesis. Underlying co-morbidities may play a role in neurological complications of CHIKV infection [5].

Reflecting the rising number of CHIKV cases since 2004, an increase in reports of vertical transmission has been noted [5]. Maternal CHIKV infection early in pregnancy does not seem to affect the fetus [10]. However, evidence of CHIKV causing neonatal disease correlates with transmission in the intrapartum period (between 2 days before and 2 days after labor), indicating that women had acute chikungunya infection near the time of delivery [10]. Previous studies showed that initial clinical features in the affected neonates included fever, distress, poor feeding, petechiae, and a maculopapular rash; all signs appearing 3–7 days after delivery [11]. However, neurological complications have been also described, and in several cases, with an impact on neonates’ long-term development, including movement, coordination, language, and sociability [11,12]. Neurological effects are not recognized at birth, but the neurocognitive function of these neonates tends to be worse compared to uninfected children [5]. 

The importance of the placenta in protecting the fetus against viral agents such as CHIKV has been demonstrated. Nevertheless, it is known that CHIKV can damage the placental tissue, altering its physiology, and thus allowing the virus passage. In addition, the consequent decrease in the oxygen exchange and nutrient transfer could contribute to fetal growth disturbances [10,13].

CHIKV is a positive-sense, single-stranded RNA virus, whose genome size is approximately 11.8 kb and it encodes two different open reading frames flanked by two untranslated regions at the 5′ and 3′ genome ends [1]. Like other RNA viruses, CHIKV’s RNA-dependent RNA polymerase is a low-fidelity enzyme and thus prone to introducing genetic variability into the viral population during each cycle of RNA replication, which ultimately allows the continuous generation of new viral variants within a single host [14]. The consequent intrahost genetic diversity is thought to be advantageous for RNA viruses by facilitating their adaptation to different environments and hosts, and might also influence their pathogenicity [15,16,17]. Furthermore, it was demonstrated for RNA viruses that just one or a few amino acid replacements within a single protein are enough to modify a particular biological characteristic of the virus [18,19]. 

Considering all the above, this project aims to assess the intrahost genetic diversity of CHIKV in infected patients with neurological manifestations, and in mother–baby pairs, i.e., women infected during the intrapartum period and their babies following vertical transmission. We explored whether the presence of particular viral variants correlated with atypical manifestations and in mother-to-baby transmission. This study enhances our understanding of these atypical CHIKV conditions, and their underlying mechanisms, ultimately contributing to better clinical management.

## 2. Materials and Methods

### 2.1. Ethical Statement

This study was approved by the Oswaldo Cruz Institute Ethical Committee in Research (CAAE 90249219.6.1001.5248 number 2.998.362). All methods were performed in accordance with the World Medical Association Declaration of Helsinki. 

### 2.2. Study Samples

Thirty samples collected from confirmed CHIKV cases in the state of Rio de Janeiro during 2019 were included in the study. Samples were referred to the arbovirus reference laboratory for diagnostic purposes, accompanied by their respective epidemiological details. Twenty samples were from ten mothers (M) and their vertically infected newborns (NB). The remaining ten corresponded to five patients with CHIKV neurological manifestations, and included cerebrospinal fluid (CSF) and serum/plasma (S/P) samples from each patient. This unique set of paired samples allows for an in depth study of intrahost diversity and intrahost selective pressure on the viral mutant ensembles in different compartments within and between hosts, in the case of vertical transmission. 

### 2.3. Viral RNA Isolation and Quantification

After an initial clarification performed by centrifuging samples at 4000× *g* over 30 min at 5 °C, total RNA was extracted from 140 µL of the supernatant using the QIAmp Viral RNA Mini kit (Qiagen, Hilden, Germany), following the manufacturer’s instructions, but with minor modifications described elsewhere [20]. Extracted nucleic acids (60 µL) were immediately treated with four units of Turbo DNase (Life Technologies, Carlsbad, CA, USA) to digest DNA contaminants, and were purified with Agencourt RNA XP beads (Beckman Coulter Genomics, Chaska, MI, USA), with a final elution in 30 µL of H2O-LA (20 µg/mL Linear Acrylamide in RNase-free water). DNased RNA (5 µL) was finally quantified by real-time RT-PCR on a 7500 Fast Real-time PCR System (Applied Biosystems, Waltham, MA, USA), following protocols described by Lanciotti et al. [21]. For the viral load estimation, a standard calibration curve was constructed using serial dilutions of RNA extracted from a CHIKV in-house viral control, with an initial concentration of 5.7 × 107 plaque-forming units (pfu)/mL. Only pairs of samples (mother + newborn or serum/plasma + cerebrospinal fluid) where a viral load ≥ 1.5 × 100 pfu/mL was detected were included in subsequent analysis. 

### 2.4. Virus Genome Deep Sequencing

Human ribosomal (rRNA) and carrier RNA were selectively depleted from DNased RNA samples, followed by randomly primed cDNA synthesis, and library construction, all performed as described previously [22], with slight differences for the depletion and cDNA synthesis depicted in Torres et al., 2021 [20]. Multiplexed libraries were quantified with a Qubit fluorometer (Life Technologies, Carlsbad, CA, USA) and analyzed with the 4200 TapeStation System (Agilent Technologies, Santa Clara, CA, USA). Finally, libraries were pooled in equimolar concentrations and paired-end sequenced in the Nextseq500 Illumina system (300 cycles) (Illumina Inc., San Diego, CA, USA).

A CHIKV-negative human serum was processed and sequenced in parallel to the samples to rule out potential cross-contamination occurring during their handling. Therefore, no CHIKV reads were expected among these control sequences. Additionally, a library was constructed from synthetic commercial plasmid pGEM-3Zf (Promega, Madison, WI, USA) to assess the potential artefactual errors introduced during libraries’ PCR amplification and sequencing.

### 2.5. Bioinformatics Data Processing

Sequencing reads were processed as described in Torres et al., 2021 [20]. Briefly, an initial trimming was carried out to remove bases and reads of low quality. Next, human sequences were depleted and reads were filtered out to retain only those belonging to CHIKV. After quality control, CHIKV reads were assembled de novo, and the representative consensus sequence of each sample was obtained. Next, CHIKV reads were mapped to their respective consensus sequence, deduplicated, and reads were realigned around insertion/deletion (indels) positions. Intrahost single nucleotide variants (iSNVs) and long variants (iLVs) representing indels, and their proportion among all CHIKV sequencing reads, were finally called by only considering alternate bases with Phred quality ≥30. Variants with significant strand bias (*p* < 0.05) or frequency lower than 1% were removed from the final dataset obtained for each sample. 

Bam files containing human depleted and CHIKV enriched sequences for each sequenced sample within this study were deposited in the NCBI Sequence Read Archive under the BioProject IDPRJNA823758.

### 2.6. Phylogenetic Analysis

Whole-genome consensus sequences obtained by deep-sequencing were aligned using MAFFT (https://mafft.cbrc.jp/alignment/server/) to 533 CHIKV sequences available in GenBank (https://www.ncbi.nlm.nih.gov; as retrieved on 12 October 2021). Sequence alignment (nucleotides 77-11313) was next manually curated to remove the artefacts. A maximum likelihood phylogenetic tree was constructed using the RaxML v8.2.12 software [23] with 1000 bootstrap replicates, under the GTR+I+G substitution model obtained by Akaike’s information criterion and likelihood value in Jmodeltest v2.1.6 [24]. The consensus tree was visualized in FigTree v1.4.4 (http://tree.bio.ed.ac.uk/software/figtree/). 

### 2.7. Viral Intrahost Genetic Diversity

To assess the potential existence of patterns of CHIKV intrahost diversity in samples in atypical and vertical transmission cases, the iSNV/LV profile obtained after variant calling was analyzed for each sample. Since the variant-calling procedure returned each minor variant’s position in the complete viral genome (nucleotides 1 to 11,811), and their specific position on each particular viral gene was computed by employing an in-house script developed in R v3.5.3 (https://www.r-project.org/). The impact of iSNV/LVs on each viral gene and encoding protein was obtained with the SnpEff software v4.3 [25]. Finally, iSNV/LV identity, frequency, position, and impact on viral proteins were assessed for each clinical condition. 

The magnitude and direction of intrahost natural selection were estimated using the ratio of non-synonymous (dN) to synonymous (dS) substitutions per non-synonymous and synonymous coding sequence sites, i.e., the standard Jukes–Cantor approach for protein-coding genes, as described elsewhere [26]. The dN/dS ratio represents a measure of selective pressure; therefore, a ratio dN/dS > 1 results when changes in the protein sequence are favored by natural selection, i.e., positively selected. On the contrary, a ratio <1 is expected if natural selection suppresses protein changes (evidence of negative selection). A dN/dS ratio equal to 1 represents a situation of neutral evolution [26]. The number of synonymous and non-synonymous sites for each sample consensus sequence was obtained with the DnaSP software v6.12 [27].

### 2.8. Analyses in Silico 

Three-dimensional (3D) protein models constructed by comparative modeling in the Swiss-Expasy server (https://swissmodel.expasy.org/, accessed on 31 October 2021) were employed to assess the structural impact of non-conservative amino acid substitutions caused by iSNVs or single nucleotide polymorphisms (SNPs; allele frequency > 50%) repeatedly identified within the samples. The models were chosen based on the global model quality estimate, the QMEAN score, the target–template sequence similarity identity percentage with templates, and crystals’ resolution and obtention method. The model’s quality was checked using the SAVES 6.0 server (https://saves.mbi.ucla.edu/, accessed on 28 November 2021). Finally, PyMol v2.4.1 was employed for visual assessment to determine how the variants under investigation could influence the structure, dynamics, and consequently function of the studied proteins.

### 2.9. Statistical Analysis

Statistical tests were performed using GraphPad Prism v8.0 (https://www.graphpad.com/scientific-software/prism/).

## 3. Results and Discussion

### 3.1. Cases Selection and Characteristics

To investigate the distribution of viral determinants potentially involved in atypical CHIKV cases or vertical transmission, we studied the intrahost CHIKV genetic diversity in samples obtained from infected mothers and their symptomatic newborns and patients with neurological manifestations. To that end, a PCR-free metagenomic experimental approach was used, intending to reflect the authentic viral intrahost diversity within each patient and between mother–baby pairs (reducing as much as possible the introduction of artifacts during the samples’ processing). Therefore, it was essential for M + NB or S/P + CSF samples to contain adequate viral RNA, as mentioned in Section 2.3 of the methodology. Only three cases of vertical transmission and three of neurological manifestations contained sufficient viral load to support further deep sequencing. Details of these cases are described below. 

#### 3.1.1. Vertical Transmission

##### Case 1 

A pregnant 24-year-old woman (M1) with uncomplicated pregnancy presented on 9 April 2019, in labor, at 38 + 4/40 weeks gestational age (GA). On admission, the patient reported arthralgia, rash, and fever of 38 °C, onset 8 April 2019. She presented with thrombocytopenia and leukopenia, which improved before being discharged from the hospital on 13 April 2019, four days after delivery. Her female newborn (NB1), born on 9 April 2019 by normal vaginal delivery, without complications, presented an appropriate weight for gestational age, Apgar score 9, and she was discharged from the hospital on her second day of life. However, on 13 April 2019 she was admitted into the Pediatric Emergency Department with fever (38.3 °C), intense crying, jaundice (+2/+6), an umbilical stump with a foul odor, and moaning. Laboratory testing showed increased C-reactive protein, thrombocytopenia, hypoglycemia, total bilirubin 9.60 mg/dL, and positive culture for E. coli. Antibiotic therapy with ampicillin 300 mg/kg and gentamicin 7.5 mg/kg was implemented for five and seven days, respectively. Ultrasonographic screening of the urinary tract showed pyelocalyceal ectasia. On 15 April 2019, a new blood sample was collected from the mother and the newborn for arboviral testing, confirming the diagnosis of chikungunya fever by positive RT-PCR testing on both samples. NB1 hospital discharge was on her 10th day of life. 

##### Case 2

A pregnant 21-year-old (M2) presented with fever and exanthema on 6 July 2019 at 36 weeks of gestational age. After testing positive for CHIKV RT-PCR on 7 July 2019, delivery was induced on 8 July 2019. Her female child (NB2) was born by caesarean section on that same day. NB2’s serum tested negative for CHIKV RT-PCR, while umbilical cord blood positive. Obstetric Doppler ultrasound and cardiotocography were normal at birth. However, on her fourth day of life, NB2 was admitted into the neonatal intensive unit, presenting apnea and seizures. On 19 July 2019, CHIKV infection was confirmed in NB2 with a positive result for CHIKV RT-PCR in a new serum sample.

##### Case 3

A pregnant 37-year-old female (M3) presented with fever (38.5), rash over the abdominal region, and arthralgia in wrists and ankles on the day of delivery (13 June 2019), at a gestational age of 36 weeks and 3 days. Her female newborn (NB3) was delivered by caesarean section due to fetal distress (altered cardiotocography). Serum samples of both M3 and NB3 tested positive for CHIKV by RT-PCR on the day of delivery. On NB3’s fifth day of life, she presented fever, maculopapular rash, and perfusion disorder, evolving with apnea, seizure, and an altered brain image on magnetic resonance. 

#### 3.1.2. Neurological Manifestations

##### Case 1

A 1-month-old male patient presented with fever, bullous lesions on his back, and a rash of one day duration. Plasma and CSF samples were taken one day after the onset of symptoms to confirm a potential arboviral infection. 

##### Case 2

A diabetic 78-year-old female patient was hospitalized after presenting with arthralgia, macular rash, fever, and confusion of 11 days’ duration (starting on 4 April 2019). Serum and CSF samples were taken on 12 April 2019 and 15 April 2019, respectively, to confirm the suspected diagnosis of arboviral encephalitis. 

##### Case 3

An 87-year-old male patient, who presented with hypertension and febrile syndrome of 4 days’ duration with intense arthralgia, petechiae, and reduced consciousness, was admitted to the hospital on 26 April 2019. His neurological symptoms progressed, but cranial computed tomography (CT) scanning was normal. The patient had suffered from viral encephalitis in the past. The presumptive diagnosis was arbovirus infection with central nervous system complications. Serum and CSF samples were collected on 30 April 2019 for laboratory confirmation. 

These different aliquots of patients’ CHIKV positive samples were processed as described in the previous section. Technical characteristics of the sequenced samples can be seen in Table S1. 

After deep-sequencing, a full-length viral consensus sequence was obtained for each sample. They were included in a dataset composed of 533 CHIKV complete genomes retrieved from GenBank. Next, the aligned sequences were phylogenetically analyzed. As expected, all sequences corresponded to genotype ECSA (Figure S1), which has been circulating in Brazil since its introduction in 2013 and was responsible for the extensive outbreak adversely affecting the state of Rio de Janeiro in 2019 (reviewed in [28]).

### 3.2. Intrahost CHIKV Genetic Diversity

**Figure 1:**
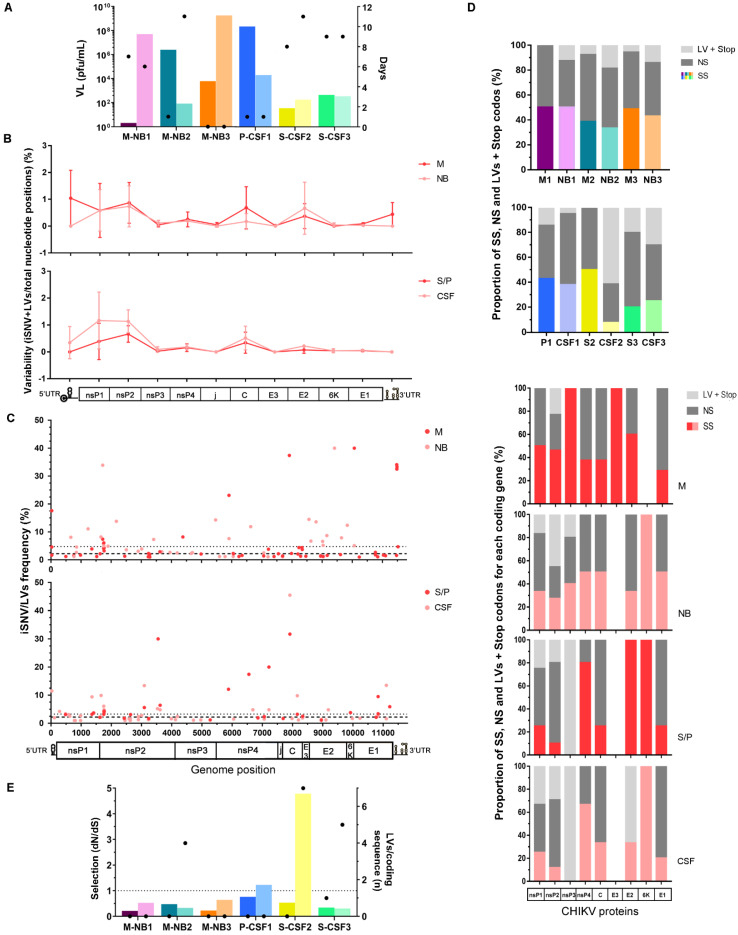
Characteristics of the intrahost viral population of cases grouped by category. (**A**) Viral load (left y-axis, represented with colored columns) and days from symptom onset up to the sample collection (right y-axis, represented with dots) for each case of the coupling samples. (**B**) Variability along the viral genome for each category. The total number of iSNVs located in each genomic region was normalized by each region’s length (total nucleotide positions). The mean value for each group (M vs. NB; S/P vs. CSF) was next calculated and plotted directly in the graph, with error bars representing the standard deviation. (**C**) iSNV/LV frequency distribution along the viral genome according to the samples’ category. Each dot represents the frequency of a single iSNV/LV, colored in dark or light red according to the group the sample belonged to. The dashed and dotted lines represent the median frequency among all iSNV/LVs found within M and NB groups (upper panel) or CSF and S/P (lower panel), respectively. (**D**) Percentage of synonymous, non-synonymous iSNVs, and LVs + stop codons. In the upper panel, the percentage frequency of each type of variant was computed for the complete coding region of each sample (each represented with a different color, following the pattern used in graph A). In the lower panel, sample categories are described separately, represented in dark or light red accordingly. The total amount of each variant class per gene was summed and then normalized by group size (n = 3) to compute next their percent frequency. Genes without graphically represented information (empty columns) indicate no variants detected in them. (**E**) Strength of natural selection acting on virus population within each host measured by the ratios of non-synonymous (dN) to synonymous (dS) substitutions per non-synonymous and synonymous coding sequence site (left y-axis, represented with colored columns) and by the accumulation of iLVs (all predicted to be deleterious) (right y-axis, represented with dots). dN/dS ratio of 1 is interpreted as evidence for neutral evolution (dotted line). dN/dS > 1 represents positive selection, while dN/dS < 1 represents a negative (purifying) selection. VL: viral load, M: mother, NB: newborn, S: serum, P: plasma, CSF: cerebrospinal fluid, nsP: non-structural protein, j: joining non-coding segment, C: capsid, E: envelope, UTR: untranslated region, SNV: single nucleotide variant, SS: synonymous variant, NS: non-synonymous variant, LV: insertion/deletion variant.

The CHIKV genomes obtained were analyzed in depth. Intrahost variants detected within each sample were filtered to retain only those with a low risk of being an error due to sample processing and NGS (no strand bias, frequency ≥1%, present in >1 read pairs) (Table S1). The ones that successfully passed through this quality control filter were aligned along the viral genome. Then, each genomic region’s total number was computed for each sample and normalized to the region’s length to obtain its variability (percent of variations per total nucleotide positions). Variability within each individual sample did not seem to follow any constant pattern. On the contrary, several genomic regions, such as nsP1, nsP2, C, and E2, seemed to present higher variability irrespective of the sample (Figure S2). However, when samples were grouped by their condition, i.e., M vs. NB or S/P vs. CSF, to check whether an emerging pattern was noted, no statistically supported difference was observed among the categories along the viral genome (ANOVA two-way tests, *p* > 0.05), including specific genomic regions such as the untranslated regions or the nsP1, nsP2, capsid, and E2 coding genes (Wilcoxon tests, *p* > 0.05) (Figure 1B). Considering the limitations of the sample size, further studies, which recruit larger numbers of cases, will be needed to address this question with sufficient power.

The intrahost frequency of every SNV/LV within each sample was also determined during the variant-calling process. The variants’ frequency was also combined with genome position to assess the frequency distribution along the genome (Figure 1C) and the median of the frequency was calculated for each group. For the vertically transmitted cases, the higher frequency of variants obtained from the NB cases (median [IQR]: 2.2 [1.4–4.4] for M and 4.8 [2.5–8.3] for NB; represented with a dashed and dotted line in Figure 1C, respectively; Mann–Whitney test, *p* = 0.018) would suggest that viral subpopulations are more represented in these cases, which could also be consistent with a potential founder effect due to a mother-to-newborn transmission bottleneck. However, it is important to note that NB median frequency could be biased by the NB2 variants’ frequency. The corresponding sample of this particular case was taken 11 days after the onset of symptoms, representing a longer viral intrahost evolutionary time period compared to the other cases. From this standpoint, and in line with previous observations of dengue-infected patients [20], it would be likely that the intrahost selective pressures may have shaped its intrahost diversity into highly representative subpopulations. Therefore, the significance of the observation of a potential effect of vertical transmission should be treated cautiously. Regarding the cases with neurological manifestations, no obvious difference was observed for the median frequency between both groups (median [IQR]: 3.2 [2.1–6.0] for S/P, 2.2 [1.5–4.8] for CSF; represented with dotted and dashed lines in Figure 1C, respectively).

We looked at the potential effect of each SNV/LV over the genome coding regions. For samples analyzed individually, overall, the proportions of synonymous (SS) and non-synonymous (NS) SNVs were similar for both samples of the pairs in all cases, except for S-CSF2 (Figure 1D). However, the NBs of the vertically transmitted cases and the CSFs of the two remaining cases with neurological manifestations presented with a higher proportion of LVs + stop codons than their paired samples (two-way ANOVA, *p* < 0.0001) (Figure 1D). Interestingly, these variants have been detected mainly in the non-structural coding genes nsP1, nsP2, and nsP3. Indels and stop codons tend naturally to create frameshifts and non-functional or truncated proteins. It is therefore possible that these resulting viral particles could be construed as “defective interfering particles” (DIPs), since their structural skeleton remains intact. As, however, their enzymes can be defective, rendering these variants less fit, we hypothesized that transmission would rely on those variants within the mutant swarm that exhibit no such mutations [29]. Particularly for the cases with neurological manifestations, LVs were also detected in the E2 coding gene within viruses from the CSF samples, while absent in the serum samples. Whether these variants were created de novo or were undetectable in serum due to very low frequency needs further exploration. DIPs have been widely described in RNA viruses and are usually associated with viral persistence and triggering antiviral immunity [30]. Therefore, our finding of DIPs needs to be considered for further studying the immune response to these defective particles at the level of the placental bed or at the blood–brain barrier (BBB). 

It is interesting to note that, besides the differences mentioned above, variation in the viral load did not present any obvious differential pattern between the categories of both groups, and interestingly, samples taken soon after the onset of symptoms, and/or at the same time (M-NB3, P-CSF1), showed a 2–3 order difference in the viral load between the samples of the pair (Figure 1A). Even though this observation involved only two cases, it raised the question of whether the viral passage through the respective barriers is a transient process occurring at an early and specific time during the infection process, and/or consistent with the unilateral direction of transmission, i.e., from the mother to neonate or from blood to the central nervous system (CNS) across the BBB. 

It has been demonstrated for several alphaviruses commonly causing viral encephalitis, such as Venezuelan and western equine encephalitis viruses (VEEV and WEEV), that passage into the CNS occurs via a caveolin-mediated transcytosis across an intact BBB [31]. However, CHIKV’s neuroinvasive phenotype is atypical [5], and the mechanisms involved in pathogenesis are still unknown. Furthermore, in vitro analyses with Semliki Forest virus (SFV), another alphavirus causing encephalitis, demonstrated that strains carrying positively charged amino acid residues at positions 162 or 247, on the surface of the E2 glycoprotein, presented a selective advantage with the ability to cross an artificial BBB, and efficiently replicate in the brain [32]. On the other hand, even though the mechanisms underlying the vertical transmission remain unknown, previous evidence suggested that CHIKV has no tropism for the placenta itself [10], limiting the chances of mother-to-child CHIKV transmission to the intrapartum period in near-term deliveries. In this context, the vertical transmission rate is close to 50% and is likely a consequence of a passive transfer of maternal blood-borne free virus particles through the placental barrier via the physiological breaches that arise at the term of pregnancy and during parturition and which are known to lead to maternal–fetal blood exchanges [33]. On the contrary, other studies demonstrated that the placenta can in fact be infected by CHIKV [34,35] and damaged as a consequence of the direct viral cytopathic effect [13]. 

In addition to the possible limited transfer through the barriers, ultimately leading to concomitant compartmentalization, is the effect of natural selection that acts within the hosts, presumably differently according to the viral location. Therefore, bottlenecks imposed after overcoming anatomical barriers and intrahost selection acting on viral variants passing through both contribute to shaping the mutant ensembles and changing variants frequencies. In this study, the natural selection strength was assessed by calculating the ratio of non-synonymous (dN) to synonymous (dS) substitutions per coding sequence site (dN/dS), considering both iSNVs and iLVs. Almost all samples (n = 10/12) presented dN/dS ratios <1 (Figure 1E), representing potential evidence that a purifying selection pressure might be taking place to shape the viral populations under study. However, two CSF cases (CSF1 and CSF2) exhibited dN/dS ratios > 1, suggesting that CHIKV was under a weaker purifying selection within these cases (Figure 1E). It should be noted that these samples were taken within 1 and 11 days since symptom onset, respectively (Figure 1A). Therefore, the time of exposure of the virus to the host does not seem to affect the strength of the purifying selection in this case; differently from what has been described for DENV-2, where prolonged infection times were in line with stronger purifying selection forces [20]. Additionally, as proposed by Grubaugh et al., the purifying selection strength was assessed by measuring the accumulation of iLVs per coding sequence too, once they are predicted to be deleterious and thus rapidly removed by selection [36]. Cases NB2, CSF2, and CSF3 showed a higher number of accumulated LVs along the sequencing coding region of the genome when compared to their predecessors.

Regarding the natural selection assessment overall, no statistically supported differences were observed for samples grouped by category (Student’s *t*-tests for dN/dS or number of LVs per coding region, *p* > 0.05 for M vs. NB, and S/P vs. CSF). Nevertheless, we hypothesized that individual evidence in NB or CSF cases could be attributable to deficiencies in the intrahost viral variants’ selection due to newborns’ immature immune responses [37] or differences between the central nervous system immunity and the peripheral one [38], respectively. Either by an obvious or subtle difference in dN/dS ratio or the amount of iLVs accumulated in the coding region, both M and S/P samples showed evidence in favor of stronger purifying selection (Figure 1E). Thus, we believe that the dynamics of the different immune responses mentioned above after overcoming anatomical barriers could explain the recovery of the viral intrahost diversity within NB or CSF samples, with the consequent appearance of defective genomes. Our hypothesis is broadly in line with previous studies demonstrating that the virus population structure could be determined by a specific tissue environment, presumably in response to tissue-specific response to infection, including innate antiviral responses [36,39]. 

### 3.3. Mutational Pattern

To study the possibility of viral factors correlating to CHIKV passage through these barriers, it is essential to trace the pattern of viral variants, if any, potentially associated with the mother/baby transmission or the neurological cases. Among all variants detected in all the samples (n = 188), 35 were repeatedly identified (Table S2). Of them, 20 were non-synonymous, and particularly, 10 involved non-conservative amino acid substitutions (Table 1). Since the latter would be expected to be potentially associated with functional changes, more detailed analyses were focused on these changes. In addition, we investigated whether there were differences between the representative consensus viral sequences of the samples (allele frequency > 50%), since they might have gone unnoticed by the bioinformatic variant-calling process used. Sixty-five different single nucleotide polymorphisms (SNPs) were identified (Table S3), of which only 14 represented amino acid substitutions, and of these only five were non-conservative (Table 1).

It is noteworthy that the relevant iSNVs and SNPs carrying non-conservative amino acid substitutions were located mainly in the non-structural coding genes, which would indicate that there is no obvious mutational pattern expressed through the viral structure that could be correlated with transmission from one compartment to the other, with the exception of the two intrahost variants found in E2 and E1 (Table 1). 

The nsP1 protein is involved in the synthesis of the viral RNA negative strand and also harbors the guanylyltransferase (GTPase) and methyltransferase (MTase) activities needed for the RNA capping [1,40]. nsP2 encodes for the viral helicase, which also acts as a protease and triphosphatase [41]. Besides its role in viral replication, its participation in pathogenic mechanisms has also been described [41,42]. nsP3 collaborates in the RNA synthesis and acts as a replicase [1], while nsP4 represents the RNA-dependent RNA polymerase [1]. E2 and E1, on the contrary, encode for the structural E2 glycoprotein, a transmembrane protein responsible for cell receptor recognition and virus-specific attachment to cells [1], and the membrane-associated E1 protein, in charge of inducing the fusion of viral and endosomal membranes, once the virus enters the cell, allowing the release of the viral nucleocapsid into the cytosol (reviewed in [43]). Therefore, considering these proteins’ crucial functions it will be essential to assess any potentially relevant effect of the substitutions described above, employing in silico analyses performed by comparative modeling.

### 3.4. In Silico Analysis of Missense Mutations

Three-dimensional models were constructed for nsP1, nsP2, and E proteins since homologous crystals were available in the PDB database (Table S4). For nsP3 and nsP4, this was not the case. Therefore, homology modeling did not result in a useful approach to analyze these proteins.

**Figure 2:**
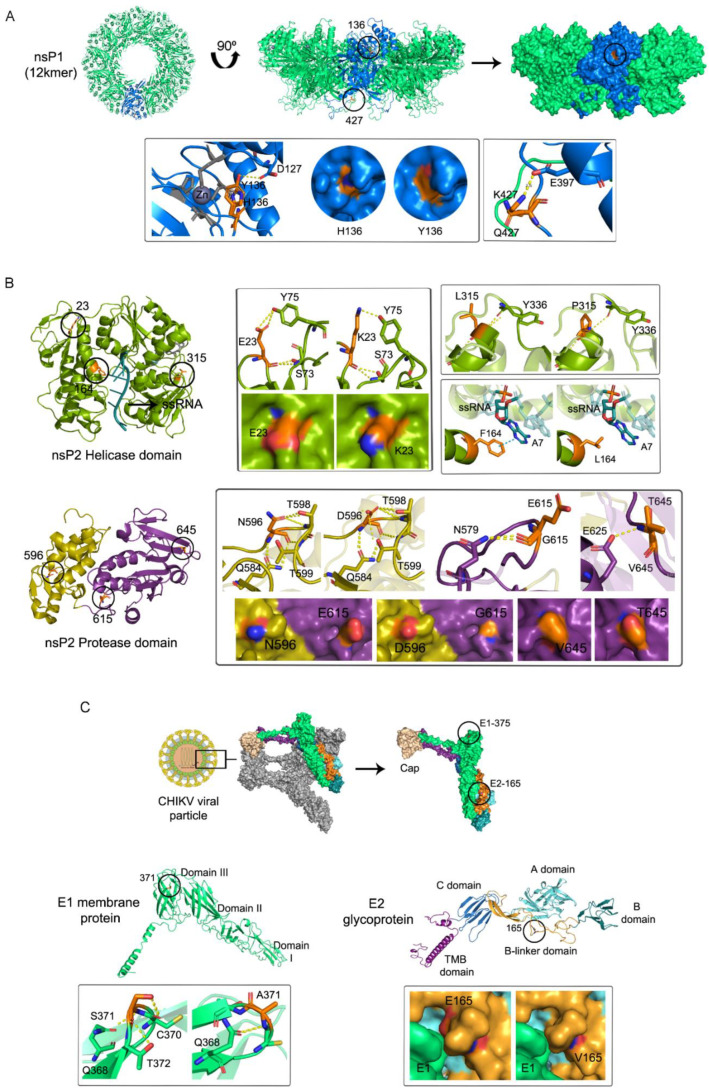
The 3D structures of CHIKV proteins. (**A**) nsP1 protein (residues 2 to 473), modeled based on crystal 7DOP. Top, its ring-shaped structure formed by 12 monomers (green) with a single subunit denoted in blue. On the left, the top view of its cartoon diagram. In the middle, the lateral view of the ring-shaped cartoon diagram, with the surface diagram on the right. The residues 136 and 427 involved in substitutions are denoted with a black circle. Bottom, side-chain interactions of residues H136, Y136, K427, and Q427. The surface disturbances caused by the former are highlighted in circles. Sticks or surface representations of these two residues are denoted in orange. (**B**) nsP2 helicase (green, residues 1–463) and protease (bottom, in yellow and purple, residues 471–791) domains, modeled based on crystals 6JIM and 4ZTB, respectively. On the left, the cartoon diagrams with the residues involved in mutations are denoted in orange and highlighted with black circles. On the right, the interactions involving these residues (orange sticks). Mutations E23K, N596D, E615G, and V645T caused electrostatic-charge shifts, evidenced on the protein surface with the red color (negative potential) representing an excess of negative charges near the surface due to oxygen’s presence, while the blue (positive potential) represents a positively charged surface, usually in line with nitrogen’s presence. (**C**) Virion surface with capsid, E1, and E2 modeled based on crystal 6NK5. Zoomed in surface and cartoon representations of E1 (green, residues 1–439) and E2 glycoproteins (residues 5–423) with its domains A (light cyan), B (dark cyan), and C (blue), connected by the beta-linker (yellow), and the transmembrane domain that connects the E2 protein with the viral membrane (purple). Bottom, cartoon representation of polar interactions involving residue S371 or A371 on E1, and surface disturbances caused by substitution E165G on E2.

CHIKV nsP1, in its active form, consists of a dodecameric ring-shaped protein (Figure 2A), with its oligomerization and the interaction with cellular membranes being essential for its enzymatic activation [40]. Minor variant H136Y was located within the capping domain (residues 1–294 and 459–472), which is structurally similar to other MTases, however, with some folding variations and the inclusion of a Zn-binding site [40]. Residue 136 was located next to this site, but its substitution from histidine to tyrosine did not disturb its architecture, nor the potential stabilizing interactions with other residues, such as D127. However, these amino acids exposed their side-chains to the protein surface, causing a local change in the relief and the electrostatic charge (Figure 2A). On the other side, the substitution K427Q compromises a residue within a disordered structure involved in membrane binding (the MBO loop-2, aa 405–430) [40]. Nevertheless, due to the lysine or glutamine side-chain orientation, the stabilizing polar contacts to other residues and the electrostatic charge over the surface remained unaltered (Figure 2A). Presumably, nsP1 interaction with the cellular membrane would also be unaffected. Finally, the effect of the substitution Q528H could not be assessed since the model prediction involved only residues 2 to 473. 

As mentioned above, nsP2 is a multifunctional protein composed of two main domains. The N-terminal domain acts as an RNA helicase, RNA-dependent 5′ triphosphatase, and nucleoside triphosphatase [41,42], while the C-terminal is a cysteine protease, which cleaves the non-structural polyprotein precursor (nsP1 to nsP4) into the different non-structural viral proteins, in a specific and sequential manner (reviewed in [44]). Three variants were mapped within the helicase domain (Figure 2B). Residue E23 is located within an unstructured loop. Through its lateral chain it interacts with S73 and Y75, presumably contributing to the structure stability. Its substitution for K23 did not disrupt these interactions but changed the net charge over the protein surface (Figure 2B). Likewise, the residue L315 is located at the end of an alpha-helix and interacts with A319 and Y336 via its main chain. Its substitution for P315 did not disturb any of these interactions nor the surface shape or electrostatic charge (Figure 2B). None of these two substitutions would be expected to alter this domain’s function. Contrarily, F164L (detected at consensus level in both the samples M2 and NB2) involved a residue compromised in RNA stabilization through stacking interactions between F164 and the RNA (Figure 2B). Docking analyses would be needed to determine if F164L could, indeed, disturb the helicase domain activity. Previous mutagenesis analyses showed that enzymes harboring mutation F164A conserved their ATPase activities, but viral replication was completely abolished [45]. From this standpoint, we hypothesize that in the case of impaired viral replication, the host antiviral response could lead to inflammatory responses and ultimately to placental damage, facilitating viral transmission. However, since this substitution was detected at the consensus level, it could be likely that any other variant might be rescuing the viral population from extinction, perhaps through epistatic effects. Otherwise, it would probably be difficult for the virus to reach a viral load in the order of 106 pfu/mL, as occurred for mother M2, or persist after 11 days of infection, as is the case of newborn NB2.

Besides the minor variant T470I for which the models built in this study failed to cover, the remaining three, N596D, E615G, and V645T, involved residues within the protease domain. Particularly, the former two were located in a random coil connector (residues 594–624) (Figure 2B), which is an unstructured linker of the two protease subdomains [44], while the latter was in a loop connecting two beta-sheets. Even though they might cause a shift in the net charge of the surface, they are not expected to disturb the labile structure of this region nor the interactions with other inner residues since they occur mainly via their main chains and not their lateral ones (Figure 2B). Consequently, they were not expected to cause any relevant disturbance to the protein function.

Lastly, only two relevant minor variants caused amino acids substitutions within the E1 and E2 glycoproteins (Table 1), which form the icosahedral shell at the virion surface (Figure 2C) [1]. Substitution E165V involved a residue located in an unstructured beta-linker subdomain, which connects the three structured and functional E2 domains (A, which contains the receptor and neutralizing antibody binding sites, B, and C) (Figure 2C) [43]. Even though E165 is a conserved residue among alphaviruses [46], this substitution did not seem to cause important alterations within the inner protein structure since the residue’s lateral chain is orientated outwards, and interactions with other residues occurred via the main chain. It neither seemed to disturb the interaction with E1, even though it slightly altered the local surface electrostatic charge (Figure 2C). Previous evidence on Semliki Forest virus (SFV), an Old-World alphavirus (*Togaviridae*), demonstrated that viruses with a positively charged lysine at E2 residue 162 or 247 were more reliant on glycosaminoglycans (GAGs) to enter cells, and therefore, more efficiently crossed an in vitro blood–brain barrier and replicated in higher titers in the brain [32]. It is unclear whether any similar scenario could be correlated to this variant, which was detected exclusively in samples of vertical transmission (Table 1).

Residue S371 was mapped to the loop connecting beta-sheets F and G, within domain III of E1 (residues 293–393) [43]. It interacted with Q385 through its main chain and C370 and T372 via its lateral chain. When mutating to alanine, the latter is interrupted (Figure 2C), however, without destabilizing this domain’s secondary structure. In addition, no obvious shift becomes evident over the protein surface, which would indicate that E1–E1 interspike contacts involving this residue would not be disrupted [43].

Finally, variants located within the UTRs need further analysis. A set of seven SNPs located within the 5′UTR was detected in two of the NB samples while absent in their respective M coupling ones, plus four S/P and CSF samples not necessarily belonging to the same patient (Table S3). Of note, these SNPs were detected in non-coupling samples, i.e., in NB or CSF samples, but not in their predecessors. In this regard, rather than suspecting a de novo intrahost origin, the most suitable explanation would be to consider a potential transmission vs. clearance balance allowing the presence/absence of these SNPs amid the coupling samples. It should be noted, however, that none of these SNPs was detected as a minor variant in any of the samples. According to the secondary structures already determined in the 5′UTR region of the viral genome [47], five of these SNPs spanning residues 7, 12, 14, 16, and 21 would participate in the stem-loop 3 (SL3) (residues 3–25), with only positions 7 and 21 being involved in base-pairing. It has been proposed that efficient viral replication relies on the SL3 structure [48]. Therefore, it should be further studied whether these variants could have any effect on viral fitness and ultimately on viral transmission from one compartment to the other. Overall, secondary structures described within the UTRs of different arboviruses seemed to be involved in viral replication but also pathogenesis (reviewed in [49,50]).

## 4. Conclusions

Even though this is a small study, the results are suggestive of a potential viral transmission event early in the course of infection, both in the case of mother–baby pairs as well as the patients with CNS involvement. Differences in the intrahost mutant swarms between the pairing samples, even when taken at the same time, indicate that viral passage through these physical barriers occurs in a particular time window and/or in a unilateral direction, and is therefore subjected to potential selective bottlenecks that shape the viral intrahost diversity. It should be better explored whether the presence of potential defective particles or replicative defective viral particles, as reported here, could be influential to the antiviral response, ultimately triggering a more compromised immune response that facilitates inflammation and placental or BBB damage, leading to vertical or blood to CNS transmission. A larger study including a variety of clinical phenotypes will be able to more comprehensively assess the role of the different variants detected here. Thus, future research should consider the potential phenotypic effects of these variants and, importantly, assess viral variants carrying conservative amino acid substitutions. Those can be detrimental as even a single substitution can modify biological properties of a virus; as is the case of the A226V mutation in E1 [18]. Our study provides a unique approach in investigating vertical transmission and CNS presentations in CHIKV infections. It can be a useful tool in exploring emerging pathogens and/or emerging new phenotypes of known pathogens. Clearly, an area critical in future preparedness.

## Figures and Tables

**Table 1:** Non-conservative iSNV/SNPs repeatedly detected in samples. Numbers in each sample’s column represent the intrahost frequency of the variant within the sample (for iSNVs), while “x” means that the variant was detected at consensus level (SNPs). M: mother, NB: newborn, P: plasma, S: serum, CSF: cerebrospinal fluid, nsP: non-structural protein, E: envelope, Nt: nucleotide, Aa: amino acid.

Region	Nt Substitution	Aa Substitution	1M	1RN	2M	2RN	3M	3R	P1	CSF1	S2	CSF2	S3	CSF3
nsP1	C482T	H136Y											3.1	3.4
	A1355C	K427Q					3.9						3.2	9.4
	G1660T	Q528H				8.2								9.9
nsP2	G1748A	E23K			4.1		5.9		3.3	4.4		6.1	3.2	
	T2171C	F164L			x	x								
	T2625C	L315P								1.0				1.6
	C3090T	T470I							5.6	13.4				
	A3467G	N596D	x	x										
	A3525G	E615G	x											
	G3614A	V645T					2.9			4.8			6.4	
nsP3	C5631T	T519M									x	x		
nsP4	C6857T	H398Y											x	x
	C7218T	T518I					3.8				20.0	4.8		
E2	A9035T	E165V				5.2	1.2							
E1	T11104G	S371A			1.5					1.6		13.5		

## Data Availability

All sequences generated in this study were deposited in the NCBI Sequence Read Archive under the BioProject ID PRJNA823758.

## Supplementary Materials

The following supporting information can be downloaded at: https://www.mdpi.com/article/10.3390/v14092006/s1, Figure S1: Maximum likelihood phylogenetic tree of DENV-2 polyprotein; Figure S2: Variability along the viral genome, plus percentage of synonymous, non-synonymous iSNVs, and LVs + stop codons on each coding gene, for individual samples; Table S1: Technical characteristics of the sequenced samples; Table S2: Variants detected repeatedly among the different samples; Table S3: Polymorphisms detected at consensus level for each sample; Table S4: Comparative modeling templates and parameters.
